# ReAS: Recovery of Ancestral Sequences for Transposable Elements from the Unassembled Reads of a Whole Genome Shotgun

**DOI:** 10.1371/journal.pcbi.0010043

**Published:** 2005-09-23

**Authors:** Ruiqiang Li, Jia Ye, Songgang Li, Jing Wang, Yujun Han, Chen Ye, Jian Wang, Huanming Yang, Jun Yu, Gane Ka-Shu Wong, Jun Wang

**Affiliations:** 1 James D. Watson Institute of Genome Sciences of Zhejiang University, Hangzhou, China; 2 Beijing Institute of Genomics of Chinese Academy of Sciences, Beijing Genomics Institute, Beijing, China; 3 College of Life Sciences, Peking University, Beijing, China; 4 UW Genome Center, Department of Medicine, University of Washington, Seattle, Washington, United States of America; 5 The Institute of Human Genetics, University of Aarhus, Aarhus, Denmark; 6 Department of Biochemistry and Molecular Biology, University of Southern Denmark, Odense M, Denmark; The National Center for Genome Resources, United States of America

## Abstract

We describe an algorithm, ReAS, to recover ancestral sequences for transposable elements (TEs) from the unassembled reads of a whole genome shotgun. The main assumptions are that these TEs must exist at high copy numbers across the genome and must not be so old that they are no longer recognizable in comparison to their ancestral sequences. Tested on the *japonica* rice genome, ReAS was able to reconstruct all of the high copy sequences in the Repbase repository of known TEs, and increase the effectiveness of RepeatMasker in identifying TEs from genome sequences.

## Synopsis

Transposable elements (TEs) are a major component of the genomes of multicellular organisms. They are parasitic creatures that invade the genome, insert multiple copies of themselves, and then die. All we see now are the decayed remnants of their ancestral sequences. Reconstruction of these ancestral sequences can bring dead TEs back to life. Algorithms for detecting TEs compare present-day sequences to a library of ancestral sequences. Unknown to many, pervasive use of whole genome shotgun (WGS) methods in large-scale sequencing have made TE reconstructions increasingly problematic. To minimize assembly errors, WGS methods must reject the highly repetitive sequences that characterize most TEs, especially the most recent TEs, which are the least diverged from their ancestral sequences (and most informative for reconstruction). This is acceptable to many, because the most important parts of the genes are not repetitive, but for the TE aficionados, it is a problem. ReAS is a novel algorithm that does TE reconstruction using only the unassembled reads of a WGS. Tested against the WGS for *japonica* rice, it is shown to produce a library that is superior to the manually curated Repbase database of known ancestral TEs.

## Introduction

Transposable elements (TEs) make up a significant proportion of many eukaryotic genomes, totaling almost 45% for human [1], and 50% to 80% for rice, maize, and wheat [2–4]. They play important evolutionary roles [5,6], and can be essential tools for genome analyses [7]. RepeatMasker (A. Smit and P. Green, unpublished data) is one of the most commonly used algorithms for detecting TEs. It relies on a comparison to known TEs from libraries like Repbase [8], which represents many years of manual labor. Computational tools like REPuter [9], RECON [10], and RepeatGluer [11] automate this process. However, almost all of the genomes sequenced today employ a whole genome shotgun (WGS) method that is incapable of assembling the most recent TEs, and any efforts to force such an assembly together generally increase the probability of assembly errors. For example, in the mouse [12] and rice [13,14] genomes, 15% and 14% of the reads were left out of the assemblies, respectively. Some of the unassembled reads are due to centromeres or telomeres, but we know in rice that many are recent TEs. Unassembled reads are the most informative reads for TE recovery as they are the least diverged from their ancestral sequences. Despite this fact, all of the above tools were only tested on assembled genomes and it is not clear how effectively or efficiently they might incorporate the information in the unassembled reads. Hence, we developed a new algorithm, ReAS (from “recovery of ancestral sequences”), to produce the requisite TE library using only the unassembled reads of a WGS.

Ancestral sequence refers to the sequence of a TE when it was first inserted in the genome, and present-day sequence refers to the sequence of a TE as it exists today. With the passage of time, all TE sequences degenerate, and after a hundred million years or so, they become unrecognizable. It is the present-day sequence that cannot be assembled by a WGS (or by ReAS), but it is the ancestral sequence that is preferred by RepeatMasker, as divergence between an ancestral sequence and a present-day copy is half of that between two present-day copies. ReAS works on TEs that satisfy two assumptions. First, these TEs must exist at high copy numbers across the genome. Second, they must not be so old that they are no longer recognizable in comparison to their ancestral sequences. For such TEs, pieces of the ancestral sequence may still exist at high copy numbers, scattered across the genome, even if nowhere in the genome is there an intact version. Reconstruction of such ancestral sequences ought to be possible, as follows.

**Figure 1:**
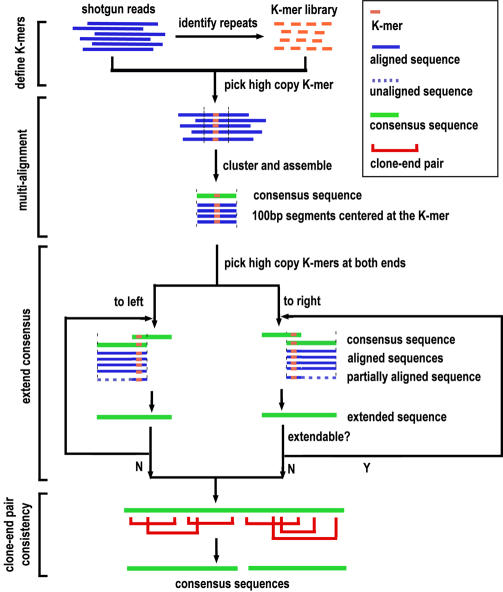
The ReAS Algorithm We start by computing *K-*mer depth, which is the number of times that a *K-*mer appears in the shotgun data. Copy number refers to how often a *K-*mer appears in the assembled genome. Depth divided by copy number is the coverage. We seed the process using a randomly chosen high-depth *K-*mer. All shotgun reads containing this *K-*mer are retrieved and trimmed into 100-bp segments centered at that *K-*mer. When the sequence identity between them exceeds a preset threshold, they are assembled into an ICS using ClustalW. We perform an iterative extension by selecting high-depth *K-*mers at both ends of the ICS and repeating the above procedure. After all such extensions are done, clone-end pairing information is used to resolve ambiguous joins and to break misassemblies, but not to join fragmented assemblies. The final consensus is our ReAS TE.

TEs are under no selective constraints once they insert into a genome. The process by which they subsequently decay is complex [15,16]. It includes mutational, insertional, and deletional events, plus transposition, amplification, and TE-mediated rearrangements. To the extent that this process is random, a consensus of present-day sequences should be a reasonable approximation of the ancestral sequence. Of course, for molecular evolution studies, a simple majority consensus is not good enough, and more detailed knowledge of the underlying biology for each TE is required to construct the correct ancestral sequence. The consensus is a good starting point, and for use as a RepeatMasker library in genome annotations, it suffices. Figure 1 depicts the ReAS process. We select a high-depth *K-*mer and retrieve all of the reads that contain this *K-*mer. Then we assemble these reads into an initial consensus sequence (ICS). Next, we look for new *K-*mers at the ends of the consensus and iteratively extend it until no further extensions are possible. Because most WGS projects generate reads from both ends of the clone inserts, we also take advantage of this linking information to resolve ambiguities and break misassemblies, but not to join fragmented assemblies. The end result is a ReAS TE.

We will demonstrate how ReAS operates on *japonica* rice, as most of the rice TEs in Repbase are from that subspecies, and unassembled WGS reads [17] are available from the GenBank trace archives. Many of the software components for ReAS were developed for RePS, the WGS assembler first used in the *indica* rice genome [18,19]. Following the nomenclature in those papers, we define the depth of a *K-*mer as the number of times that it appears in the unassembled data. Copy number is how often it appears in the assembled genome. Shotgun coverage is the ratio of depth to copy number, which for *japonica* is 6×. The essential difference between RePS and ReAS is that the former avoids high-depth *K-*mers, and the latter seeks them out. These contrasting objectives are fundamentally at odds with each other. RePS must choose between leaving behind a lot of unassembled reads, versus having larger contigs with potentially more assembly errors. ReAS focuses on TE recovery alone, and is therefore more likely to get the right answer, in contrast to the other reconstruction algorithms like REPuter, RECON, or RepeatGluer, which must operate on a genome that is already misassembled by algorithms like RePS. All C subroutines and Perl scripts for ReAS are freely available from ReAS@genomics.org.cn.

## Results

One of the luxuries of doing this analysis on *japonica* is the fact that we have data from Syngenta [17], which uses a WGS method, and from the International Rice Genome Sequencing Project (IRGSP) [20–22], which uses a mapped-clone method. ReAS was run on the unassembled WGS reads from Syngenta, but when an assembled genome sequence was needed, we used the IRGSP results. The recovery process was seeded with *K-*mers of length *K* = 17, and a depth threshold of *D* = 14 was used. Mitochondria and chloroplast sequences were removed before analyzing the resultant ReAS TEs. The “gold standard” against which we benchmarked the recovered TEs was Repbase version 8.4.

### Repbase Comparison

**Figure 2:**
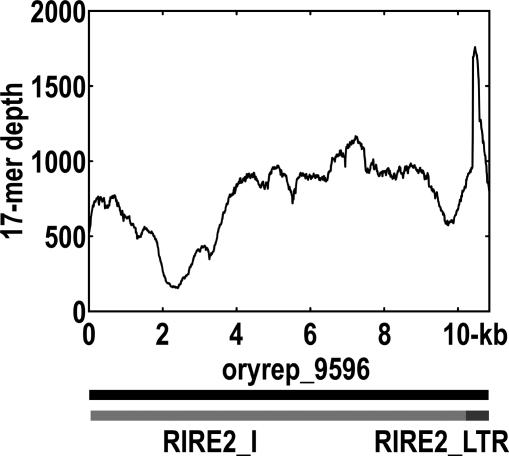
Complete Recovery of Known TE RIRE2 is a *gypsy*-like TE that is found in two pieces in Repbase, as RIRE2_I (Internal) and RIRE2_LTR (LTR). These have 96% and 93% nucleotide identity to our ReAS TE, respectively.

**Figure 3:**
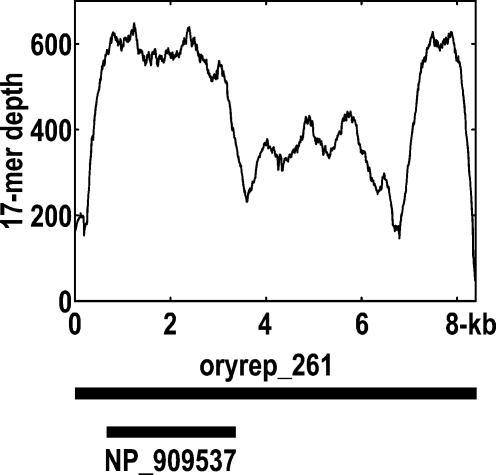
Recovery of Unknown TE Although not found in Repbase, we believe that this ReAS TE is a valid reconstruction, because it has a BlastX match with identity 98% over 869 amino acids to a TE-related protein (gi|34896386|ref|NP_909537.1| Putative mutator like transposase) that is annotated in a GenBank clone.

Figure 2 is an example of a perfectly recovered TE that exists in fragmented form in Repbase. This *gypsy*-like element is 10,841 bp. The region from 1 to 10,387 bp matches Repbase RIRE2_I (Internal) at 96% nucleotide identity, and the region from 10,401 to 10,841 bp matches Repbase RIRE2_LTR (long terminal repeat [LTR]) at 93% nucleotide identity. Notice that ReAS only recovered the LTR at one end of this TE, even though the correct ancestral sequence should have an LTR at both ends. This is a consequence of our restriction that reads already in a consensus be excluded from the next extension. Figure 3 depicts a recovered TE that had no counterpart within Repbase. We believe it is a valid TE because it has a BlastX alignment at 98% identity over 869 amino acids to a TE-related protein in GenBank (gi|34896386|ref|NP_909537.1| Putative mutator like transposase).

More than 95% of the 17-mers in the recovered ReAS TEs had depths over 14, as we show in Figure S1. This is of course by design. In contrast, for the Repbase TEs, only half of the 17-mers had depths over 14. This is relevant for the comparisons, because not every one of these low-depth Repbase TEs is correct. It is clear, however, that ReAS cannot recover them, so for the comparisons, we considered only high-depth TEs in Repbase. WGS reads were aligned to Repbase TEs using BlastN. Good alignments were those of size greater than 100 bp and nucleotide identity of better than 85%. High-depth TEs were covered over 80% of their length with at least 14 aligned reads. A total of 54 Repbase TEs qualified, and these are described in Table S1. As we show in Table 1, 95.8% of 54 high-depth Repbase TEs were recovered, although sometimes in fragmentary form. If fragmentary recovery is not acceptable, and we credited only the best-matched ReAS TEs, 88.1% were recovered. The reduction in the recovery rate is mostly attributable to *copia* elements, which tend to have lower 17-mer depths and more divergent sequences.

**Table 1:**
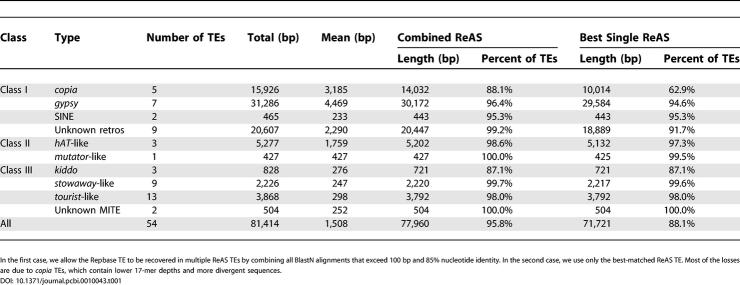
Nearly Complete Recovery of All 54 High-Depth TEs in Repbase

Table S2 compares each of these Repbase TEs to its best-matched ReAS TE. Over aligned regions, sequence identities averaged 96.8%. Where they failed to align, we computed error rates. False negative (FN) is the fraction of the Repbase TE that remains unaligned. It averages 11.9% in our dataset. False positive (FP) is the fraction of the ReAS TE that remains unaligned, but we must make some exceptions because there are many plausible explanations for these unaligned regions. We know that Repbase can be incomplete. ReAS TEs were larger than Repbase TEs in 42 of 54 cases. In seven instances, ReAS TEs were 2–18 times larger than Repbase TEs. This was due either to incomplete Repbase TEs, in the manner of Figure 2, and/or to concatemers of the form |- LTR -| |- Internal -| |- LTR -| |- Internal -|, which tend to occur when the LTRs are extremely diverged. Ignoring these seven instances, the average ratio of ReAS TE to Repbase TE size was actually 1.01. For our definition of FP, therefore, we ignored any unaligned regions at the ends and counted only unaligned regions in the middle, as these are the problems that are most likely to mislead a user. The average FP over the dataset was 1.6%.

### Utility as TE Library

ReAS recovered 8,411 high-depth ancestral sequences with mean length of 640 bp and mean depth of 152, as we indicate in Table 2. Of these, 1,275 matched to known TEs in Repbase and 707 matched to TE-related proteins (keywords include retrotransposon, transposase, reverse transcriptase, *gypsy,* and *copia*); the remaining 6,429 were less easily classified. This last category is primarily composed of small low-depth repeats. Indeed, they drag down the overall mean length and depth. If we included only those repeats that matched to Repbase, the mean length was 1,634 bp and the mean depth was 464. Based on the arguments that we discuss below, we further subdivided this last category into 1,792 potentially interesting repeats (of length over 500 bp and depth over 35) and 4,637 probable artifacts.

**Table 2:**
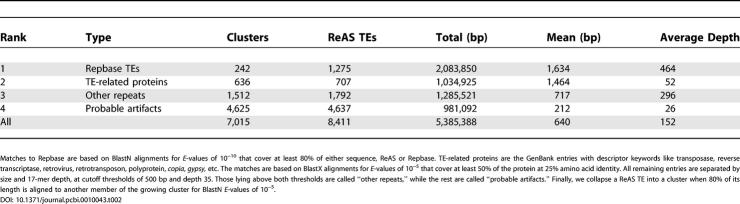
Ranking of All 8,411 ReAS TEs Based on Their Likelihood of Being TEs

Many of the ReAS TEs could be clustered together, on the criterion that a repeat was collapsed into a cluster when 80% of its length aligned with another member of that cluster at BlastN *E*-values of 10^−5^. This reduced the dataset to 7,015 clusters. The collapse was most pronounced among repeats with a match to Repbase, where 1,275 ReAS TEs were collapsed into 242 clusters. To explore the extent to which highly divergent TEs are assembled into different ReAS TEs, we performed a simulation. We started with an ancestral sequence of 500 bp, 2 kb, and 10 kb. From this, we simulated 100 present-day copies of the TE, with a range of divergences from the ancestral sequence. We simulated a 6× WGS and applied ReAS to that. Results were averaged over ten such simulations, as shown in Table S3. Some fragmentation was observed in the more divergent TEs, especially at larger sizes. However, even in the worst case of 0% to 40% divergence at 10 kb size, a single best-matched ReAS TE covered 86% of the ancestral TE. All of the other pieces were smaller than 500 bp in size and less than 35 in depth. Some collapsed into the best-matched ReAS TE. Almost all aligned to the ancestral TE, of which we invariably recovered more than 95%.

Table 3 classifies the 1,275 known TEs in the same manner as our previous *indica* genome analyses [13,14]. We recovered 691 (in 113 clusters) Class I TEs. There were 51 (in 25 clusters) *copia* and 381 (in 41 clusters) *gypsy* LTR retrotransposons, relatively few LINEs (long interspersed nuclear elements) and SINEs (short interspersed nuclear elements), plus 239 (in 39 clusters) unknown retrotransposons. This outcome is consistent with the finding that LTR retrotransposons are the single largest component of most plant genomes [23]. The ratio for *copia* to *gypsy* elements was 51/381 (or 25/41 by clusters). This is consistent with the finding that *copia* is less abundant than *gypsy* [24]. We recovered 217 (in 48 clusters) Class III TEs. These were typically quite small, with a mean size of 396 bp, and found in noncoding regions adjacent to genes [25].

**Table 3:**
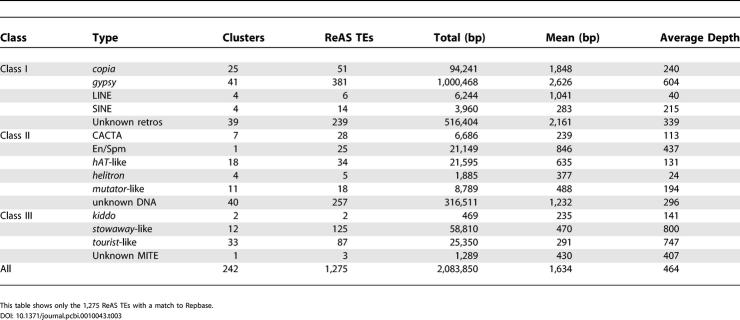
Classification of Recovered Sequences into Known TE Families

We subdivided the unclassified repeats by comparing their characteristics with those of our 1,275 known TEs. If we set a threshold at depth 35, then 9.3% of known TEs fell below this threshold, as opposed to 4,637 (84.7%) of 5,475 unclassified repeats of length under 500 bp. Conversely, we created chromosome-sized random sequences of length 10 Mb, simulated a 6× WGS on these, and ran ReAS. From ten such chromosomes, 3,419 ReAS TEs were recovered. These were obviously artifacts, which was easy to see because only 31 (0.9%) had length over 500 bp, while 38 (1.1%) had depth over 35. As a result, we used these cutoff thresholds to clean up our 6,429 unclassified repeats. Of the remaining 1,792 repeats, 89 (in 29 clusters) were of length over 1,000 bp and depth over 100, as shown in Tables S4–S9. One cluster of 45 repeats was centromeric in origin. Two clusters were attributable to ribosomal RNA and a seed prolamine gene. To check for pseudogenes, we searched for similarity to a nonredundant set of 19,079 full-length cDNAs [26] that are called nr-KOME. Seven clusters matched at BlastX *E*-values of 10^−5^, but five of these seven matched to cDNAs that show similarity to recently discovered TE-related proteins. If we eliminate these, we are left with 23 repeats (in 19 clusters) of mean length 1,795 bp and mean depth 739. This is comparable to known TEs. All but two of these clusters are 80% intact in the Syngenta or IRGSP assemblies, indicating they are not ReAS artifacts.

**Figure 4:**
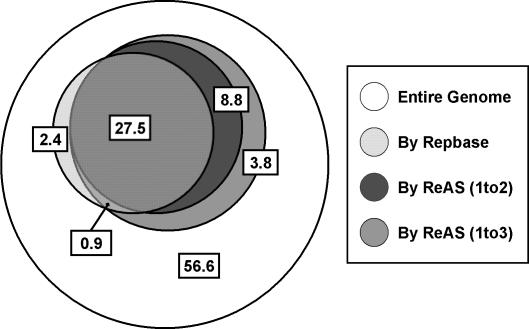
Masking of Entire Rice Genome by RepeatMasker We use different TE libraries and indicate the overlaps between these different results in a Venn diagram. ReAS (1to2) refers to the first two categories of Table 2 (Repbase and TE-related). ReAS (1to3) includes the third category (other repeats). Numbers are percentage of genome.

The ultimate test of utility is to use these ReAS TEs as a library for RepeatMasker and see how well that masks the rice genome. We did this analysis on the IRGSP genome, because this sequence was taken by a mapped-clone method, and is more representative of the true repeat content. Figure 4 and Table 4 show the comparison to and improvement over Repbase. Only 30.8% of the genome was masked using Repbase as the library, whereas 36.3% was masked when we used the ReAS TEs in the known and TE-related categories. This increased to 41.0% if we added the third category of “other repeats.” To consider whether gene duplications were a confounding factor, we ran RepeatMasker on the 19,079 gene regions defined by nr-KOME cDNAs. Only 1.7% of the nucleotides for the coding exons were masked, but 9.3% were masked if introns were included. In fact, even those few percent that were masked are not likely due to gene duplications, as there are TEs in cDNAs. Of the 246 genes where more than half of the coding exons were masked, four were ribosomal RNAs and the rest were BlastP homologous to TE-related proteins at *E*-values of 10^−5^.

**Table 4:**
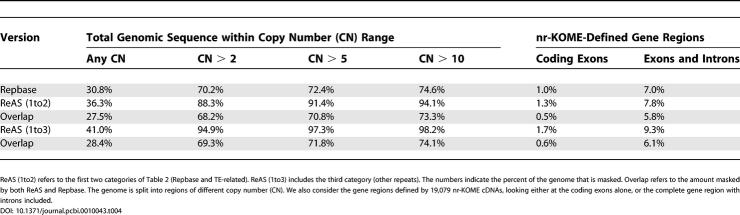
Application of RepeatMasker to the Entire Rice Genome, Using Different Versions of the Repeat Library

## Discussion

Given the inherent complexity of the ReAS algorithm, it is astonishing how well it does work, especially when benchmarked against the years of skilled labor that went into the production of Repbase. We would caution that our parameters were specifically tuned for rice, and they will likely need to be changed for non-grass genomes, to adjust for how TE history is different in other species. Even in rice, our current parameters worked much better for *gypsy* than for *copia* TEs. ReAS works best for high-copy TEs that have not had too much time to diverge from their ancestral sequences. The same constraints apply even in a manual reconstruction procedure, and a lot of hard work will be required to do much better than ReAS. Although it is true that Repbase has many low-depth TEs that are not in ReAS, there is often no evidence that these Repbase entries represent the correct ancestral sequence of any particular TE. Indeed, by using ReAS as the library in RepeatMasker, we can detect many more TEs than Repbase while missing little of what is in Repbase. Some manual curation is required for the post-analysis of recovered TEs. For example, |- LTR -| |- Internal -| |- LTR -| |- Internal -| concatemers must be resolved manually. ReAS removes a lot of the drudgery, but it is not the final answer. What it does is provide a starting point for expert annotation of the complete TE contents of a sequenced genome.

## Materials and Methods

### The ReAS algorithm.

We explain our choice of parameter settings in a later section. Here, we wish to discuss the principles behind the ReAS algorithm. In this paper, *K* = 17. Selection of the initial *K-*mer seed must satisfy three conditions. First, to avoid spurious matches, it cannot be a simple repeat like a poly-A tail. Second, the *K-*mer depth must exceed a predetermined threshold *D*. In this paper, *D* = 14. At a coverage of 6×, this corresponds to a copy number of 2.3. Finally, it should not be in a previously recovered ReAS TE.

Using the selected *K-*mer as a bait, we retrieve all sequence reads that contain this *K-*mer, and trim the reads down to 100-bp fragments centered at that *K-*mer. We align the fragments with each other and look for groups of *D* or more fragments with what we call 95% “mutual identity.” That is, we add fragments successively, and at each step, ask that the new fragments be 95% identical to at least 2/3 of the pre-existing fragments in that group. To avoid the combinatorial explosion, we employ a greedy algorithm. For the initial seeding, we consider only the biggest resultant group. For the extensions, we must consider all the resultant groups. This will create ambiguity problems. How these are resolved is discussed in a later section. Two commonly used alignment tools were tested: ClustalW [27] and Phrap (P. Green, unpublished data). ClustalW excelled at assembling a large number of fragments of somewhat differing sequence content, while Phrap excelled at assembling a small number of fragments of nearly identical sequence content. ClustalW tolerated the inevitable discrepancies, while Phrap often failed to assemble fragment groups as a result of these discrepancies. We therefore chose ClustalW.

To extend the consensus, we select high-depth *K-*mers from both ends of the ICS, and use these as secondary seeds to retrieve additional reads. Also, to reduce the chances that something might be missed, because of mutations or rearrangements, we consider all qualified *K-*mers (i.e., those satisfying the above seed conditions) within 50 bp of the ends. We require that the newly retrieved reads agree with the previous ICS in this 50-bp region, to within 95% identity. A constraint is imposed on the number of shared reads *b*. Suppose there are *a* + *b* reads in the previous ICS, and *b* + *c* reads in the new consensus. We ask that *b*/(*a* + *b*) > 20% or *b*/(*b* + *c*) > 20% or *b* > 200. Assuming that these conditions are satisfied, we extend the ICS by100 bp, starting from the end of the previous ICS. Since most reads are 500 bp long, no read is allowed to participate in more than five extensions. This process is repeated until no further extensions are possible, after which, a finishing step is used to get the last few bases. We walk out one base at a time, and stop whenever we see a 20-bp region where fewer than 60% of the reads agree to 95% mutual identity.

### General difficulties.

The idealized algorithm described above is of course a simplification. In practice, there are three problems: ambiguity/misassembly, fragmentation, and segmental duplication. To resolve ambiguities and break misassemblies, we consider the long-range information that is available to us. The reads themselves at typical lengths of 500 bp are one source of such information. Use of read overlap data is implicitly incorporated, because we ask that the parent ICS and its extension share some portion of their reads. Another source of such information arises when reads are sampled from both ends of the clone inserts, which are typically 3 kb apart. Consider the following example.

**Figure 5:**
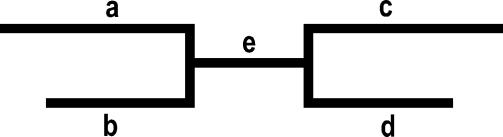
Fork Problem during Consensus Extension Suppose we have two TEs, each in three segments, a-e-c and b-e-d, where segment e is identically shared between the two TEs. Four results are possible (a-e-c, a-e-d, b-e-c, and b-e-d), and ReAS will compute all four paths. It then uses the overlapping reads or clone-end pairs for bridging information, and where possible, eliminates any incorrect paths.

Figure 5 shows a situation where two distinct TEs, a-e-c and b-e-d, share a similar fragment, e. Four reconstructions are possible: a-e-c, a-e-d, b-e-c, and b-e-d. If the shared region is not too long, for example, less than300 bp, the correct path may be identified by read overlaps. If not, the correct path may still be identified by clone-end pairing data. A few possibilities are listed here: (1) if a-e-c and b-e-d are supported, and nothing else, the other two paths can safely be eliminated; (2) if a-e-c is the only supported path, it is a less certain situation, but since the other path is most likely to be b-e-d, these are the paths we keep; (3) if a-e-c and a-e-d are both supported, it is difficult to know which path is correct, and so we keep all four; (4) if none of the four paths are supported, we keep them all. The operating philosophy is that we try to resolve all the ambiguities as safely as possible, but if it cannot be done, we keep all possible paths for future consideration.

**Figure 6:**
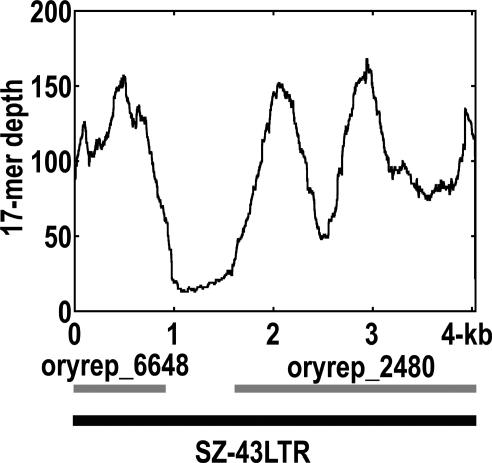
Fragmentation due to Low *K-*mer Depth SZ-43LTR is the LTR region from a TE that is found as one piece in Repbase, but is recovered by ReAS as two nonoverlapping pieces, with 98% and 97% nucleotide identity to the Repbase entry.

Some TEs will not be recovered as a single consensus if, at some point along their length, the depth falls below *D*. The example in Figure 6 is for a *gypsy-*like TE called SZ-43LTR. Of the two recovered consensus fragments, one covers positions 1 to 927 bp and, compared to the Repbase TE, shows 98% nucleotide identity. The other covers positions 1,568 to 4,039 bp and shows 97% nucleotide identity. As expected, the break is in a region of low depth. Although in principle one can use clone-end pairing data to join fragmented assemblies, in practice we discovered that this procedure is too error-prone. For example, two distinct TEs may be adjacent to each other on the genome, and therefore they will be linked by a clone-end pair. Hence, we decided not to use the clone-end pairing data in this context. The information lost turns out to be minimal.

**Figure 7:**
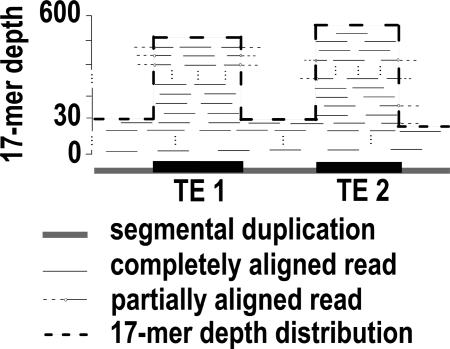
TEs within Segmental Duplications If the duplication is of sufficiently high copy number, it will be assembled as a “ReAS TE,” and what we need to do afterwards is find the boundaries of the TEs within this assembled duplication. On the assumption that TEs have much higher copy numbers, TE boundaries can be identified by sudden changes in depth, accompanied by many partially aligned reads.

Segmental duplication of large regions of a genome [28] causes another problem, because when the duplication is of sufficiently high copy number, it will be assembled as a “ReAS TE.” To the extent that there are TEs inside this duplication, we must determine their boundaries. We use an idea from RECON [10], taking advantage of the fact that TEs tend to occur at much higher copy numbers than duplications. Figure 7 explains the basic concept. TE boundaries are identified by sudden changes in depth, accompanied by many partially aligned reads. For any particular read, we define the endpoint as the boundary of its alignment with the ReAS TE. We search for any regions with a significant aggregation of endpoints and a significant depth discrepancy. On the low side, the depth is required to be less than 300. We ask that the ratio of high to low depth be greater than three. On the high side, we also require that there are endpoints for at least 50% of reads within 20 bp of the putative boundary. TEs so defined are then excised for further analysis.

### Parameter settings.

In principle, ReAS is applicable to any genome (not just rice), with the appropriate changes in parameter settings. Table 5 lists the settings used for the Syngenta *japonica* 6× WGS, and explains how they might be adjusted for other genomes. For the specialists, we discuss the technical issues here. Consider the choice of *K*. It must be large enough for 4*^K^* to exceed the genome size. *K* ≥ 15 suffices for rice. Our algorithm needs a byte of memory for every possible *K-*mer, so there is a limit to how large *K* can be. Sixteen gigabytes are used at *K* = 17. Notice also that larger *K*s might make it more difficult to recover the older TEs, where only the smallest fragments are still recognizable.

**Table 5:**
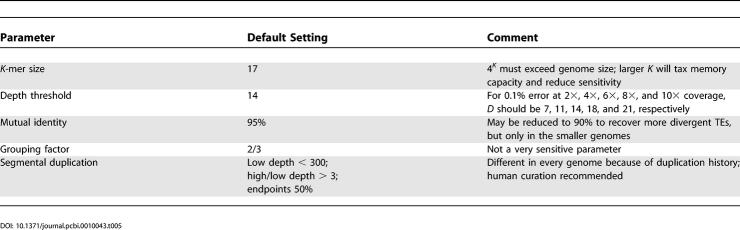
Default Parameter Settings for Rice WGS

The threshold depth *D* is selected based on coverage and error rate considerations. If we assume that, in the unique portions of the genome, the read depths follow a Poisson distribution, then for a nominal coverage of 6× and a threshold depth of *D* = 14, one would expect a 0.1% probability for a unique sequence to be mistakenly called repetitive. This is of course only an approximate guideline, as the read depths do not really follow a Poisson distribution. As we show in Figure S2, 32.4% of the *K-*mers have a depth of one, because of sequencing errors. The number is large because a single error in one base pair will ruin every *K-*mer that overlaps with it. The data were filtered for base pairs of error probability worse than 10^−2^, but it is possible that our error probability is too optimistic, since we did not control the experimental conditions, and calibration was difficult.

We decided on the 95% mutual identity rule after a process of trial and error. For more divergent TEs in smaller genomes, a less stringent rule may be used. We would not recommend that it be less than 90%, because of the increased likelihood of misassemblies and the increased strain on computational resources. The 2/3 grouping factor is not a sensitive parameter. We simulated 1,000 present-day sequences by mutating an ancestral sequence to give divergences of 0% to 10%. The greedy algorithm treats each sequence as a node. Arcs are placed between every pair of nodes that pass the mutual identity threshold. We select the node with the most arcs, and consider all the other nodes in succession. The rule is that, when the number of arcs to the cluster exceeds the grouping factor, that node is added to the cluster. The number of nodes per cluster does vary with mutual identity, but at a 95% setting, it varies only 11% for grouping factors of 1/2 to 4/5.

For the extension process, we require that the number of shared reads exceed 20% or 200 reads. In theory, if all of the TE copies are full length, 80% of the reads should be shared because most reads are 500 bp and the ICS grows by 100 bp at a time. In practice, the 20% rule is easily satisfied by over 99% of valid (i.e., based on comparison to known TEs) reconstructions. However, without some sort of shared reads criterion, we would get too many misassemblies. The 200-reads threshold is not a sensitive parameter. Both parameters are robust, and we would not change either of them regardless of the genome.

We benchmarked parameters for the RECON-inspired splitting against segmental duplications from the assembled rice genome. We used known TEs to determine the read depth and breakpoint distributions. Reads that belong to the duplication are fully aligned, but those that belong to a TE from somewhere else are only partially aligned and break at the TE boundary. One such example is shown in Figure S3. In practice, we would expect the situation to be different in every genome, reflecting differences in duplication history, and these parameters must be adjusted accordingly.

## Supporting Information

Figure S1 Comparison of Repbase and ReAS Showing Amount of Dataset at the Stated 17-mer Depth Amount of data is defined by the total length of the TEs, not the number of TEs, so longer TEs contribute more to the ordinate. The vertical line marks our *D* = 14 threshold.
(593 KB TIF)

Figure S2 Observed Distribution of 17-mer Depths for Syngenta WGS versus Expected Poisson Distribution at Shotgun Coverage of 6× Observed values are indicated by a solid line; expected values are indicated by a dashed line.
(560 KB TIF)

Figure S3 Example of a TE in a Segmental Duplication This duplication has five copies in the entire genome, but living inside it is a TE with hundreds of copies. Reads are aligned in BlastN. Hits must exceed 100 bp and 85% nucleotide identity. Endpoints are declared when a read has over 50 unaligned bases at the end.
(578 KB TIF)

Table S1 Description of All 54 High-Depth TEs in Repbase We compared shotgun reads to Repbase TEs. BlastN alignments that exceed 100 bp and 85% nucleotide identity are mapped back to the Repbase TEs. High-depth TEs are those that are over 80% covered by high-depth alignments (HD blocks) of depth over 14.
(20 KB XLS)

Table S2 Best-Matched ReAS TE for All 54 High-Depth TEs in Repbase Asterisks have been appended to the names of those seven outliers that are clearly due to incomplete Repbase TEs and/or LTR concatemers. We define FN as the fraction of a Repbase TE that remains unaligned. Conversely, FP is the fraction of a ReAS TE that remains unaligned, where we ignore unaligned regions at the ends and count only those in the middle. Total and average are given in the final row. For the size ratio, we exclude the seven outliers. For FP and FN, we compute length-weighted averages.
(29 KB XLS)

Table S3 Effects of TE Sequence Divergence We started with an ancestral sequence of 500 bp, 2 kb, and 10 kb. From this, we simulated 100 TE copies with the stated range of divergences from the ancestral TE. We simulated a 6× WGS and ran ReAS. Results are averaged over ten such simulations. This table indicates the number of recovered ReAS TEs, the number of clusters they collapse into, the number of likely artifacts of size less than 500 bp and 17-mer depth less than 35, the percentage of ancestral TE covered by all ReAS TEs, the percentage covered by only the best-matched ReAS TE, and the number of recovered pieces that cannot be aligned to the ancestral sequence.
(18 KB XLS)

Table S4 Description of Known ReAS TEs We show length, mean 17-mer depth, cluster number, and classification information.
(228 KB XLS)

Table S5 Description of TE-Related ReAS TEs We show length, mean 17-mer depth, cluster number, and classification information.
(134 KB XLS)

Table S6 Description of Other ReAS TEs We show length, mean 17-mer depth, cluster number, and classification information. Here, we also consider non-TE interpretations by indicating (under “class”) if the entity is a simple or low-complexity repeat, and (under “notes”) if it is a ribosomal RNA or centromeric repeat. To check for likely pseudogenes, we searched for similarity to the nr-KOME cDNAs using BlastX at an *E*-values of 10^−5^. To verify that what we recovered is not an artifact of the ReAS process, we indicate the number of intact copies found in the Syngenta (WGS) and IRGSP (clone-by-clone) assemblies, where by “intact” we mean 80% of the ReAS TE is aligned at 85% nucleotide identity.
(238 KB XLS)

Table S7 FASTA-Formatted Sequence for Known ReAS TEs (2.1 MB TXT)

Table S8 FASTA-Formatted Sequence for TE-Related ReAS TEs (1.0 MB TXT)

Table S9 FASTA-Formatted Sequence for Other ReAS TEs (1.3 MB TXT)

## Abbreviations

FN: false negative
FP: false positive
ICS: initial consensus sequence
IRGSP: International Rice Genome Sequencing Project
LTR: long terminal repeat
TE: transposable element
WGS: whole genome shotgun
